# FNDC5 attenuates obesity-induced cardiac hypertrophy by inactivating JAK2/STAT3-associated inflammation and oxidative stress

**DOI:** 10.1186/s12967-019-1857-8

**Published:** 2019-04-02

**Authors:** Zhi Geng, Wen-Yong Fan, Bing Zhou, Chao Ye, Ying Tong, Ye-Bo Zhou, Xiao-Qing Xiong

**Affiliations:** 1 Key Laboratory of Cardiovascular Disease and Molecular Intervention, Department of Physiology, Nanjing Medical University, 101 Longmian Avenue, Nanjing, 211166 Jiangsu China; 2 Department of Cardiac Surgery, The Second Affiliated Hospital of Nanjing Medical University, Nanjing, 210011 Jiangsu China; 3 State Key Laboratory of Medical Neurobiology, Department of Physiology and Biophysics, School of Life Sciences and Collaborative Innovation Centre for Brain Science, Fudan University, Shanghai, 200438 China

**Keywords:** FNDC5, Cardiac hypertrophy, Obesity, Inflammation, Oxidative stress

## Abstract

**Background:**

Chronic low-grade inflammation and oxidative stress play important roles in the development of obesity-induced cardiac hypertrophy. Here, we investigated the role of Fibronectin type III domain containing 5 (FNDC5) in cardiac inflammation and oxidative stress in obesity-induced cardiac hypertrophy.

**Methods:**

Male wild-type and FNDC5^−/−^ mice were fed normal chow or high fat diet (HFD) for 20 weeks to induce obesity, and primary cardiomyocytes and H9c2 cells treated with palmitate (PA) were used as in vitro model. The therapeutic effects of lentiviral vector-mediated FNDC5 overexpression were also examined in HFD-induced cardiac hypertrophy.

**Results:**

High fat diet manifested significant increases in body weight and cardiac hypertrophy marker genes expression, while FNDC5 deficiency aggravated cardiac hypertrophy evidenced by increased *Nppa*, *Nppb* and *Myh7* mRNA level and cardiomyocytes area, in association with enhanced cardiac inflammatory cytokines expression, oxidative stress level and JAK2/STAT3 activation in HFD-fed mice. FNDC5 deficiency in primary cardiomyocytes or FNDC5 knockdown in H9c2 cells enhanced PA-induced inflammatory responses and NOX4 expression. Exogenous FNDC5 pretreatment attenuated PA-induced cardiomyocytes hypertrophy, inflammatory cytokines up-regulation and oxidative stress in primary cardiomyocytes and H9c2 cells. FNDC5 overexpression attenuated cardiac hypertrophy as well as cardiac inflammation and oxidative stress in HFD-fed mice.

**Conclusions:**

FNDC5 attenuates obesity-induced cardiac hypertrophy by inactivating JAK2/STAT3 associated-cardiac inflammation and oxidative stress. The cardio-protective role of FNDC5 shed light on future therapeutic interventions in obesity and related cardiovascular complications.

**Electronic supplementary material:**

The online version of this article (10.1186/s12967-019-1857-8) contains supplementary material, which is available to authorized users.

## Background

Cardiac hypertrophy has become an independent and predictive risk factor for adverse cardiovascular events [1]. Despite an increasing understanding toward the pathophysiological process of cardiac hypertrophy, progress in mechanism remains sluggish in recent years. Many factors are involved in the pathophysiological process of cardiac hypertrophy, of which inflammation and oxidative stress play crucial mediating role [2, 3]. Among cardiovascular diseases, obesity has a great impact on the cardiac remodeling and function in terms of hemodynamic load, myocardial fibrosis and impaired ventricular contractility, which leads to the progression of heart failure [4, 5]. Obesity has also been characterized as an inflammatory state [6, 7], and chronic low-grade inflammation and oxidative stress play important roles in the pathogenesis of obesity-related complications. It has been demonstrated both in vitro and in vivo that the increased free fatty acids in obesity can activate the nuclear factor-kappaB (NF-kB) pathway, subsequently increasing the expression of several pro-inflammatory cytokines and inducing cellular oxidative stress [8]. However, the precise molecular mechanisms involved in obesity-induced cardiac hypertrophy process still need further elucidation.

It is well established that fibronectin type III domain containing 5 (FNDC5) and its cleaved and secreted fragment, irisin, are beneficial to improve metabolic diseases in both humans and mice [9]. Recent studies in our lab have shown that FNDC5 overexpression ameliorates hyperlipemia and enhances lipolysis in adipose tissues of mice [10], and prevents high fat diet (HFD)-induced hyperlipemia, hepatic lipid accumulation and impaired fatty acid oxidation in liver [11]. Irisin improves glucose homoeostasis by reducing gluconeogenesis and increasing glycogenesis via PI3K/Akt pathway in HepG2 cells [12]. These findings of our previous studies confirmed the beneficial effects of FNDC5/irisin in metabolic diseases. Many studies have highlighted the role of exercise in the heart, of which serves as an important organ regulating metabolism. Exercise training has various physiologic effects on the cardiovascular system, most notably multiple metabolic changes in the myocardium resulting in improved tolerance for ischemia and reperfusion injury, a reduction of resting heart rate attributed to increased parasympathetic tone and an improved vascular endothelial function with augmented flow-mediated vasodilation during exercise [13–15]. Nevertheless, as an up-regulated myokine after exercise, the exact role of FNDC5 in cardiomyocytes remains unclear.

Growing evidence points to the regulatory capacity of JAK2/STAT3 pathway on inflammation-related diseases and cardiac hypertrophy [16–19]. STAT3 is the most important effector of the JAK2 in the initiation and development of cardiac hypertrophy. After phosphorylation, STAT3 translocates from the cytoplasm to the nucleus and promotes the transcription of multiple hypertrophic factors including natriuretic peptide type A and natriuretic peptide type B [20]. Therefore, it is worthy to examine whether JAK2/STAT3 pathway is involved in molecular mechanisms of obesity-induced cardiac hypertrophy. We recently reported the anti-inflammatory effects of FNDC5 in adipose tissue of mice [21]. Here, the aim of this study is to evaluate the role and molecular mechanism of FNDC5 on cardiac function, inflammatory and oxidative stress responses in HFD-induced obese mice, which would be used for future therapeutic interventions in obesity-related complications.

## Methods

### Animals

Male wild-type (WT) mice and FNDC5^−/−^ mice on a C57BL/6 background (Nanjing BioMedical Research Institute of Nanjing University, Nanjing, China) were used in the study as previously reported [11]. Mice were housed at 22–26 °C temperature, 40% ± 5% humidity and 12-h light/dark cycle. At the age of 6 weeks either WT or FNDC5^−/−^ mice were randomly divided to fed a normal chow (Ctrl, 14.7 kJ/g, 13% of energy as fat) or a HFD (21.8 kJ/g, 60% of energy as fat) for 20 weeks, respectively. The lard-based HFD were obtained from Nanjing Junke Biotechnology Corporation, Ltd (Nanjing, China).

### FNDC5 overexpression in mice

As we previously described, WT and FNDC5^−/−^ mice at the age of 6 weeks were fed with HFD for 20 weeks to induce obesity. FNDC5 overexpression were induced by lentivirus (1 × 10^8^ TU/ml, 100 μl) intravenously injection [10]. The recombinant lentivirus expressing FNDC5 or vector were injected into the mice at the end of the 14th week after HFD. Acute experiments were carried out 6 weeks after the lentivirus injection.

### Echocardiographic measurements

Cardiac function was measured by echocardiography (Vevo 2100, VisualSonic, Canada). WT and FNDC5^−/−^ mice were anesthetized using 2.0% isoflurane and then two-dimensional echocardiographic views of the mid-ventricular short axis were obtained at the level of the papillary muscle tips below the mitral valve. Interventricular septal thickness (IVS), left ventricular internal dimensions (LVID) and left ventricular posterior wall dimensions (LVPW) were measured at the end of diastole and systole [22].

### Western blot analysis

Tissues or cardiomyocytes were sonicated in RIPA lysis buffer containing 1% NonidetP-40, 0.1% SDS, protease inhibitor, while adding phosphatase inhibitors cocktail (Bimake, Houston, USA), and then homogenized. The nuclear and cytosolic protein were extracted by commercially available kit (Beyotime Biotechnology, Shanghai, China). Briefly, the cells expanded sufficiently under low osmotic pressure, then the cell membrane ruptured, releasing cytoplasmic proteins. Nucleus precipitation could be centrifuged to obtain after then. Finally, the nuclear protein was extracted by high salt nucleoprotein extraction reagent. Equal quantities of tissues or cardiomyocytes protein lysates were subjected to SDS-PAGE (Bio-Rad), and transferred onto PVDF membrane. Blocking was made at room temperature with 5% nonfat milk powder prepared in Tris-buffered saline containing 0.1% Tween 20. Then, membranes were incubated overnight at 4 °C with corresponding antibodies, and followed by incubation with appropriate secondary HRP-conjugated antibodies. Antibodies against p38 (#8690), P-p38 (#4511), NFκB-p65 (#8242), ERK (#4695), P-ERK (#4370), P-Stat3 (#9145), Stat3 (#4904) and GAPDH (#2118) were obtained from Cell signaling Technology (Danvers, USA). Antibodies against FNDC5 (ab174833), P-JAK2 (ab195055), JAK2 (ab108596), Nox2 (ab129068), Nox4 (ab154244) were obtained from Abcam (Cambridge, UK).

### Quantitative real-time PCR analysis

Total RNA was separated using a Trizol reagent (Life Technologies, USA) according to the manufacturer’s protocols. RNA concentrations and purity were assessed by the measurement of optical density at 260 and 280 nm. The purified total-RNA was reverse transcribed with reverse transcription reagent kit (Takara, Japan) and quantitative real-time PCR was performed on Stepone Plus system using the SYBR Green Master Mix (Takara, Japan). The fold changes in the genes were analyzed based on comparative ΔΔCt method. The sequences of primers were listed in Additional file 1: Table S1.

### Tissue embedding and H&E staining

The mice were anesthetized and the heart were quickly removed. Heart tissues were rinsed in ice-cold PBS, and incubated overnight in formalin. After washed and dehydrated by successive incubation in 70 to 100% ethanol solutions, heart tissues were fixed and embedded in paraffin, and slices were cut every 6 μm. The sections were stained with hematoxylin–eosin (H&E) and used for histopathological analysis. The degree of pathological changes was evaluated microscopically by measuring the cardiomyocyte area.

### Cell culture and treatment

Neonatal primary cardiomyocytes (CMs) from 1 to 3-day-old WT or FNDC5^−/−^ mice were prepared as reported [23]. Briefly, neonatal hearts were dissociated in cell suspension using the Neonatal Heart Dissociation Kit (MACS, Bergisch Gladbach, Germany). Dissociated cells were neutralized with DMEM and 4.5 g/l glucose supplemented with 7.5% FBS and passed through a 70-µm cell strainer. Then the dissociated cells were preplated at 37 °C for 1 h to remove fibroblasts and endothelial cells, and nonadhered cells were collected as CMs. CMs were plated in DMEM supplemented with 15% FBS, 1% penicillin, and 1% streptomycin in a humidified atmosphere of 5% CO_2_ at 37 °C. Embryonic rat heart-derived cell line H9c2 were obtained from Costar Corning Inc. (Corning, CA, USA). H9c2 was cultured in DMEM/F12 medium containing 2.25 g/l glucose medium supplemented with 10% FBS, 1% of penicillin, and 1% of streptomycin. CMs and H9c2 cells were treated with palmitate (PA, obtained from Sigma Aldrich) at 400 μM (bound to BSA at a ratio of 5:1) for 24 h to mimic high lipid stimulation and to induce inflammation. PA was administrated 4 h after exogenous FNDC5 (200 nM, Sigma Aldrich) pretreatment. For WP1066 (5 μM, Med Chem Express) treatment experiments in H9c2 cells, WP1066 was added 2 h before FNDC5 pretreatment, while PA was added 4 h after FNDC5 pretreatment. The measurement for phosphorylated protein level was made 12 h after PA administration, while for mRNA levels or protein levels were made 24 h after PA administration.

### Small interfering RNA transfection

H9c2 cells were pre-treated with specific small interfering RNA (siRNA) against FNDC5 (FNDC5-siRNA). The FNDC5 specific siRNA (sense sequence: 5′GAUGGCCUCUAAGAACAAA3′; antisense sequence: 3′CUACCGGAGAUUCUUGUUU5′) was purchased from RiboBio (Guangzhou, China). Negative siRNAs (NA-siRNA) with no sequence homology to any known rat genes were used as a control. H9c2 cells were transfected with 50 nM siRNA using lipofectamine 3000 reagent (Invitrogen). Cells were lysed, 48 h after transfection, to analyze FNDC5 protein expression to examine knock-down efficiencies.

### Oxidative stress detection

The heart tissues or cells were washed and added to phosphate-buffered saline, ground into homogenates, and centrifuged to collect the supernatant. The activities of superoxide dismutase 1 (SOD) and the content of malondialdehyde (MDA) were detected by commercially available kits purchased from Nanjing Jiancheng Bioengineering Institute (Nanjing, China). Griess Reagent Kit (Thermo Fisher Scientific, Waltham, USA) was used to determine the estimation level of Nitric Oxide (NO) production in cell culture medium of CMs according to the manufacturer’s protocol.

### ELISA

TNF-α, IL-1β levels in heart tissues were measured with mouse TNF-α and IL-1β ELISA Kits (Excell Bio, Shanghai, China). IL-6 levels in heart tissues were measured with mouse IL-6 ELISA Kit (Beyotime Biotechnology, Shanghai, China). ELISA kit for FNDC5 was obtained from Phoenix pharmaceuticals (EK-067-19, Burlingame, USA).

### Statistical analysis

All values were presented as mean ± SEM. Student’s t-test was used for 2-group comparisons. Experiments with > 2 groups were compared by 1-way or 2-way ANOVA followed by Bonferroni’s multiple comparisons test. Statistical analyses were performed with SPSS software. A value of P < 0.05 was considered statistically significant.

## Results

### FNDC5 deficiency worsened HFD-induced cardiac hypertrophy in mice

**Fig. 1:**
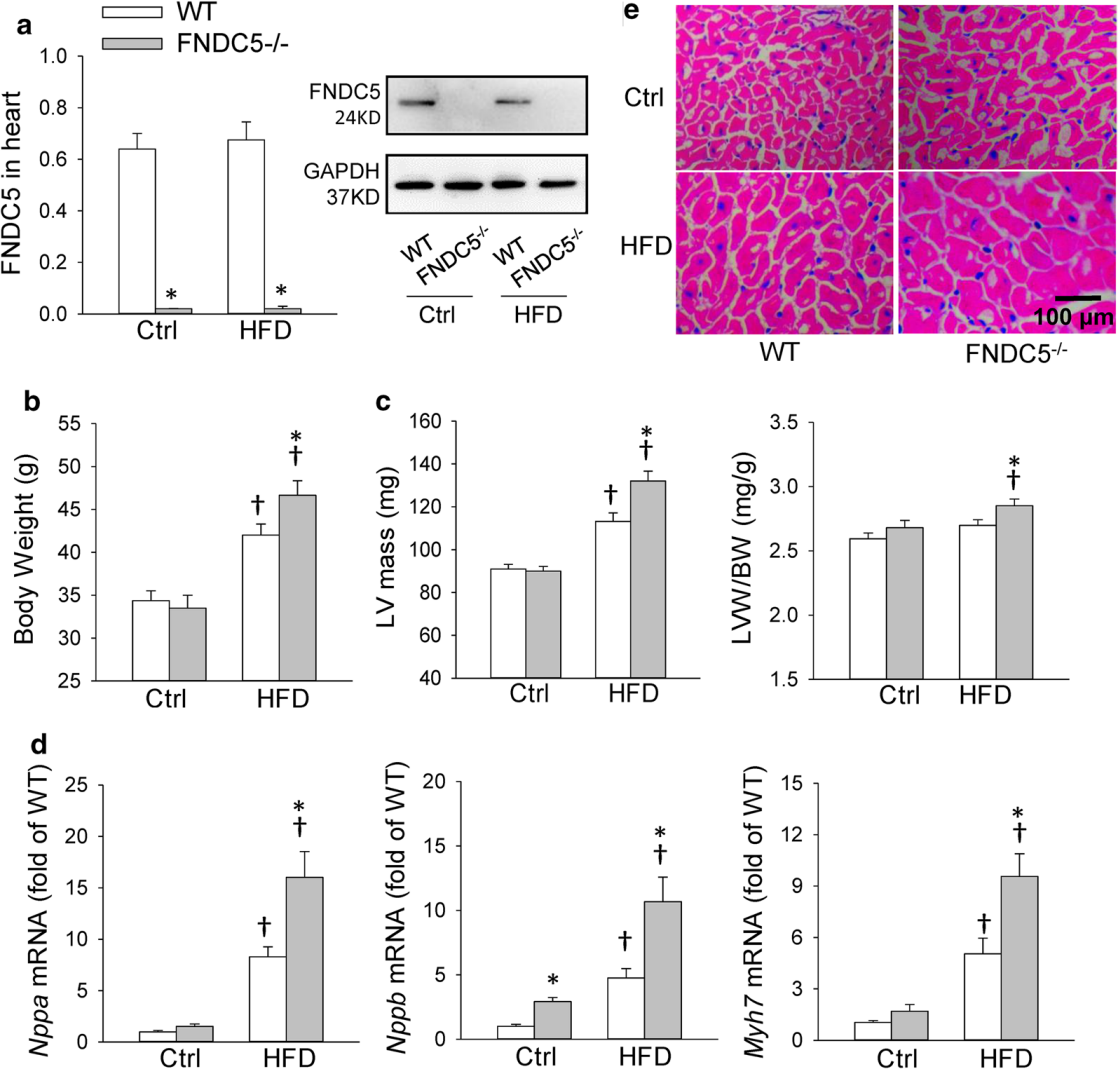
FNDC5 deficiency worsened HFD-induced cardiac hypertrophy in mice. WT and FNDC5^−/−^ mice were fed with mouse chow diet (Ctrl) and high fat diet (HFD) for 20 weeks. The measurements were carried out at the end of 20 weeks after Ctrl or HFD feeding. **a** Expression of FNDC5 protein in heart determined with Western blot. **b**, **c** Body weight (BW), left ventricular (LV) mass and left ventricular weight/body weight (LVW/BW). **d** The mRNA expression of cardiac hypertrophy markers in heart. **e** Representative sections of H&E staining show the cardiomyocyte area in heart. Values are mean ± SEM. *P < 0.05 vs. WT. ^†^P < 0.05 vs. Ctrl. n = 6

FNDC5^−/−^ mice were used to determine the roles of FNDC5 in HFD-induced myocardial hypertrophy in the present study. We have identified the efficiency of FNDC5 deficiency in our recent study, and we further confirmed that FNDC5 was deleted in heart of mice in the present study (Fig. 1a). However, HFD feeding did not significantly change FNDC5 protein expression in heart. FNDC5 gene deletion aggravated HFD-induced obesity, which was consistent with our recent study [21] (Fig. 1b). Echocardiographic analysis showed that, despite lack of an obvious difference in left ventricular ejection fraction (LVEF) and fractional shortening (LVFS) among HFD-fed WT and FNDC5^−/−^ mice, FNDC5 deficiency significantly augmented left ventricular hypertrophy evidenced by increased IVSd and LVPWd and cardiac hypertrophy markers (*Nppa, Nppb, Myh7*) mRNA expression as well as LVW/BW (Table 1, Fig. 1c, d) after HFD treatment. Moreover, HFD feeding significantly increased cardiomyocyte diameter as demonstrated by H&E staining, and FNDC5 gene deletion further aggravated it in HFD-fed mice (Fig. 1e, Additional file 1: Figure S1A). Taken together, these results indicate that FNDC5 deficiency was associated with deterioration in myocardial hypertrophy in response to HFD.

**Table 1:** Echocardiographic assessment of left ventricle functions in mice

	Ctrl-WT	Ctrl-FNDC5^−/−^	HFD-WT	HFD-FNDC5^−/−^
IVSd (mm)	0.7 ± 0.02	0.74 ± 0.02	0.75 ± 0.01^†^	0.82 ± 0.02*^†^
IVSs (mm)	1.07 ± 0.02	1.1 ± 0.03	1.19 ± 0.02^†^	1.20 ± 0.02^†^
LVIDd (mm)	4.05 ± 0.11	4.0 ± 0.11	3.98 ± 0.14	4.01 ± 0.1
LVIDs (mm)	3.16 ± 0.13	2.98 ± 0.1	2.73 ± 0.12^†^	2.84 ± 0.08
LVPWd (mm)	0.72 ± 0.02	0.74 ± 0.03	0.77 ± 0.02	0.85 ± 0.02*^†^
LVPWs (mm)	1.17 ± 0.02	1.18 ± 0.03	1.25 ± 0.02^†^	1.24 ± 0.03
LVEF (%)	59.4 ± 2	57.3 ± 1.6	48.1 ± 1.7^†^	49.7 ± 1.7^†^
LVFS (%)	32.2 ± 1.3	30.3 ± 1.2	25.2 ± 1.2^†^	24.7 ± 1.5^†^

*IVSs and IVSd* interventricular septum at end systole and diastole, *LVIDs and LVIDd* left ventricular internal dimensions at end systole and diastole, *LVPWs and LVPWd* left ventricular posterior wall dimensions at end systole and diastole, *LVEF* left ventricular ejection fraction, *LVFS* left ventricular fractional shorting

Values are mean ± SEM. *P < 0.05 vs. WT. ^†^P < 0.05 vs. Ctrl. n = 6

### FNDC5 deficiency aggravated cardiac inflammation in HFD-induced obesity

**Fig. 2:**
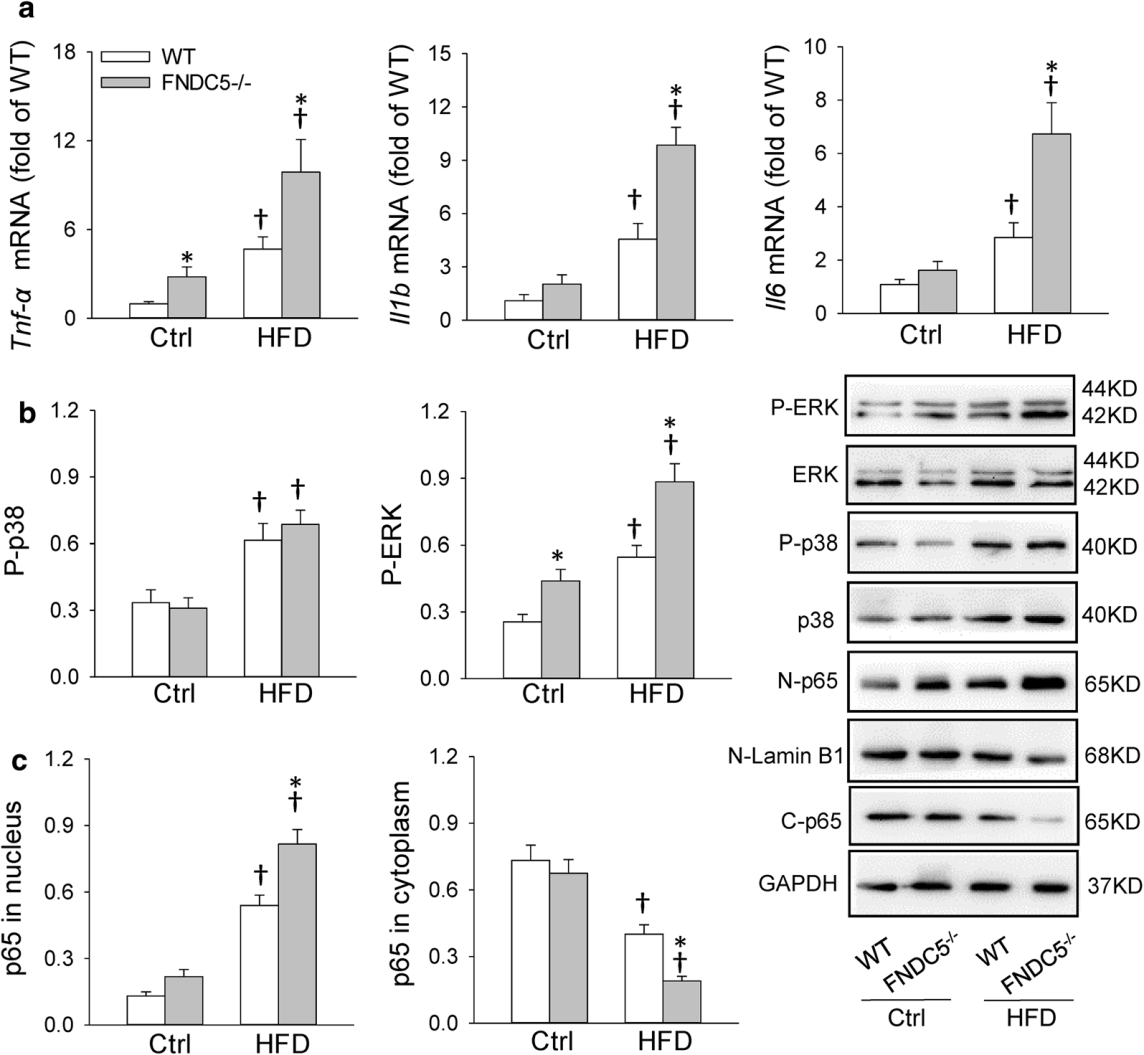
FNDC5 deficiency aggravated HFD-induced inflammation in heart. **a** Cardiac *Tnf*-*α, Il1b* and *Il6* mRNA levels determined with qPCR. **b** Phosphorylation levels of p38 and ERK in heart determined with Western blot. **c** NFκB activation in heart. N-p65: p65 in nucleus; C-p65: p65 in cytoplasm. Values are mean ± SEM. *P < 0.05 vs. WT. ^†^P < 0.05 vs. Ctrl. n = 3

As we known, HFD causes substantial inflammatory responses in the heart, which mediates the pathogenesis of cardiac injury and remodeling. We firstly examined the expression of pro-inflammatory cytokines in heart. *Tnf*-*α, Il1b* and *Il6* mRNA levels were upregulated after HFD, while FNDC5 gene deletion exacerbated the enhancement of them (Fig. 2a). However, FNDC5 deficiency had no significant effects in mice with control diet. The expression of cardiac inflammatory cytokines were also determined with ELISA (Additional file 1: Figure S1B–D). It is established that NLR family pyrin domain containing 3 (NLRP3) inflammasome activation were associated with increased inflammatory mediators and profibrotic factor production, resulting in myocardial fibrosis, cardiomyocyte hypertrophy and impaired cardiac function [24]. Here, we also examined the gene expression of NLRP3 inflammasome and downstream inflammatory mediators IL-18 in heart. As shown in Additional file 1: Figure S2, HFD caused an up-regulated levels of *Nlrp3* and *Il18* mRNA, while FNDC5 deficiency further aggravated the enhancement of mRNA levels.

On the other hand, NFκB is a primary regulator of inflammatory responses, which induces the expression of pro-inflammatory cytokines in the heart [25]. NFκB activation and inflammation signals included phosphorylation of p38 mitogen-activated protein kinase and p44/42 mitogen-activated protein kinase (ERK) were also examined herein. The NFκB activation indicated by increased p65 in nucleus and reduced p65 in cytoplasm, and the enhanced phosphorylation level of ERK suggested that FNDC5 deficiency aggravated cardiac inflammation in obesity. However, there was no significant change in phosphorylation level of p38 between WT and FNDC5^−/−^ mice fed with control diet or HFD (Fig. 2b, c).

### FNDC5 deficiency aggravated cardiac oxidative stress in HFD-induced obesity

**Fig. 3:**
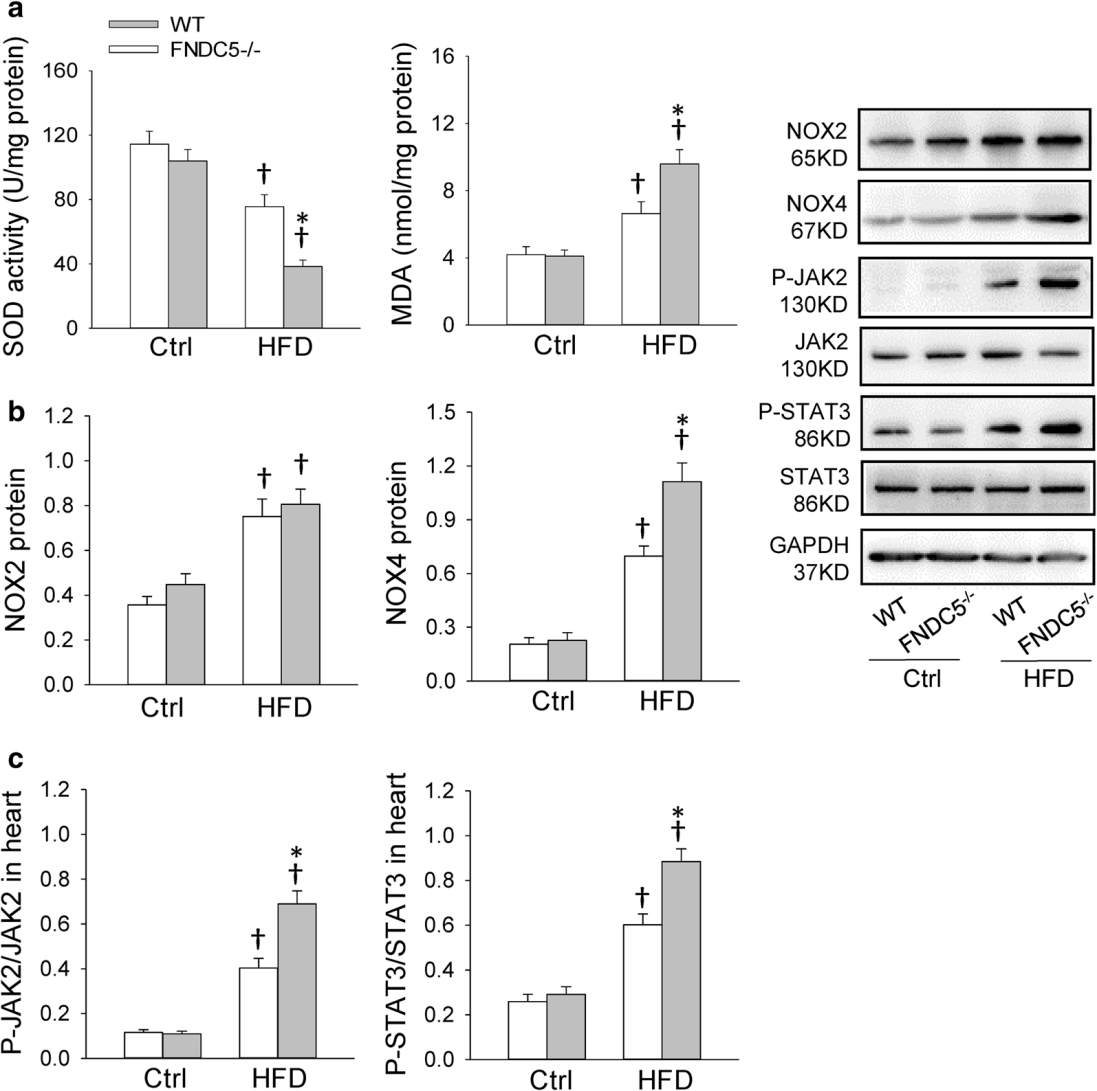
FNDC5 deficiency enhanced HFD-induced oxidative stress and phosphorylated JAK2 and STAT3 level in heart. **a** Superoxide dismutase (SOD) activity and malondialdehyde (MDA) level in heart. **b** Expression of NAPDH oxidases (NOX2 and NOX4) protein in heart. **c** Phosphorylated JAK2 and STAT3 level in heart. Values are mean ± SEM. *P < 0.05 vs. WT. ^†^P < 0.05 vs. Ctrl. n = 6

Studies suggested that hyperlipidemia can hyperpolarize mitochondria causing electron slippage and enhanced ROS production, leading to cardiac injury and obesity-related complications [26]. The imbalance between ROS generation and the antioxidant system occurs due to a change in the overall redox balance and in the modification of target molecules, resulting in inflammation [27]. Free radical scavenging enzymes, SOD, can eliminate excessive ROS during the cardiac injury. MDA is a biological marker of oxidative stress to determine the degree of the peroxidation of membrane lipid. The decreased SOD activity and increased MDA level caused by HFD were further exacerbated by FNDC5 gene deletion (Fig. 3a). Nicotinamide adenine dinucleotide phosphate (NADPH) oxidase are dominant mechanisms of ROS production in heart. Among the NADPH oxidase isoforms, NOX2 and NOX4 are abundantly expressed in cardiomyocytes [28]. As shown in Fig. 3b, HFD led to increased expression of NOX2 and NOX4, while FNDC5 deficiency further enhanced NOX4 level but not NOX2. These results suggest that FNDC5 is involved in obesity-induced cardiac oxidative stress.

### FNDC5 deficiency/knockdown aggravated palmitate-induced inflammation in primary cardiomyocytes and in H9c2 cells

**Fig. 4:**
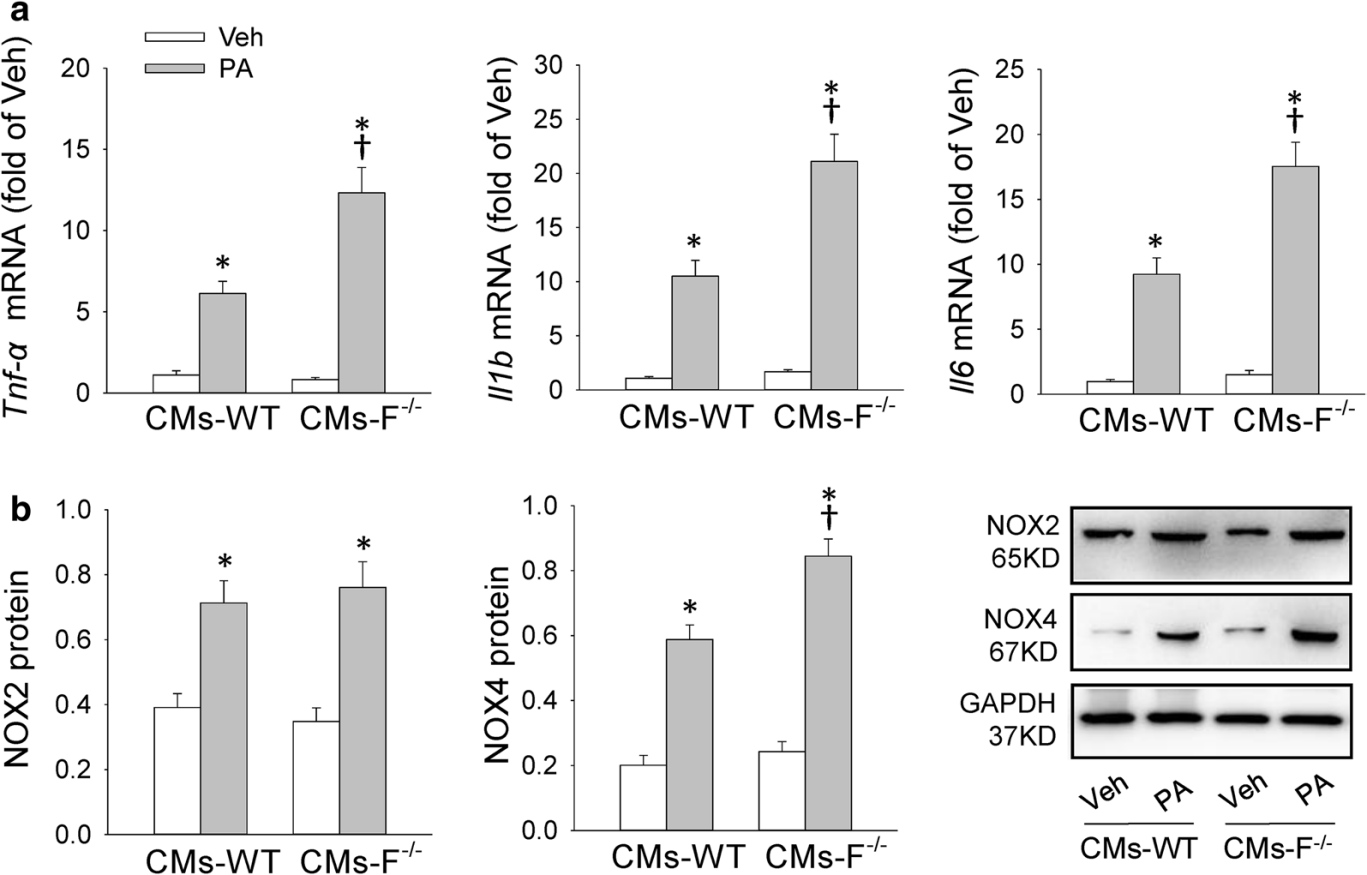
FNDC5 deficiency enhanced palmitate-induced inflammation and NOX4 expression in primary cardiomyocytes (CMs). **a** Effects of palmitate (PA, 400 μM) treatment on *Tnf*-*α, Il1b* and *Il6* mRNA levels in CMs of WT and FNDC5^−/−^ mice. **b** Effects of PA treatment on NOX2 and NOX4 protein level in CMs of WT and FNDC5^−/−^ mice. The measurement was 24 h after PA treatment for measuring the mRNA levels or protein levels. PA: Palmitate; Veh: Vehicle; F^−/−^: FNDC5^−/−^. Values are mean ± SEM. *P < 0.05 vs. Veh. ^†^P < 0.05 vs. CMs-WT. n = 3

The in vivo findings urged us to further determine the effects and mechanisms of FNDC5 on inflammation and oxidative stress in vitro. We used neonatal primary cardiomyocytes (CMs) and H9c2 cells treated with palmitate (PA) to mimic fatty acid stimulation. CMs derived from WT and FNDC5^−/−^ mice and H9c2 cells with FNDC5 siRNA silencing were used to determine the role of FNDC5 in hyperlipidemia-induced cardiomyocytes damage. We observed that FNDC5 deficiency in CMs enhanced PA-induced *Tnf*-*α, Il1b* and *Il6* mRNA levels augmentation as well as upregulated expression of NOX4 but not NOX2 (Fig. 4a, b). Also, transfection of si-FNDC5 significantly reduced FNDC5 protein expression in H9c2 cells, which further led to an increased gene expression of *Tnf*-*α, Il1b* and *Il6* as well as NOX4 protein in H9c2 cells (Additional file 1: Figure S3). These results confirmed the important role of FNDC5 in high lipid induced cardiomyocyte hypertrophy in vitro.

### Exogenous FNDC5 pretreatment attenuated palmitate-induced cardiomyocyte hypertrophy, inflammation and oxidative stress in primary cardiomyocytes and in H9c2 cells

**Fig. 5:**
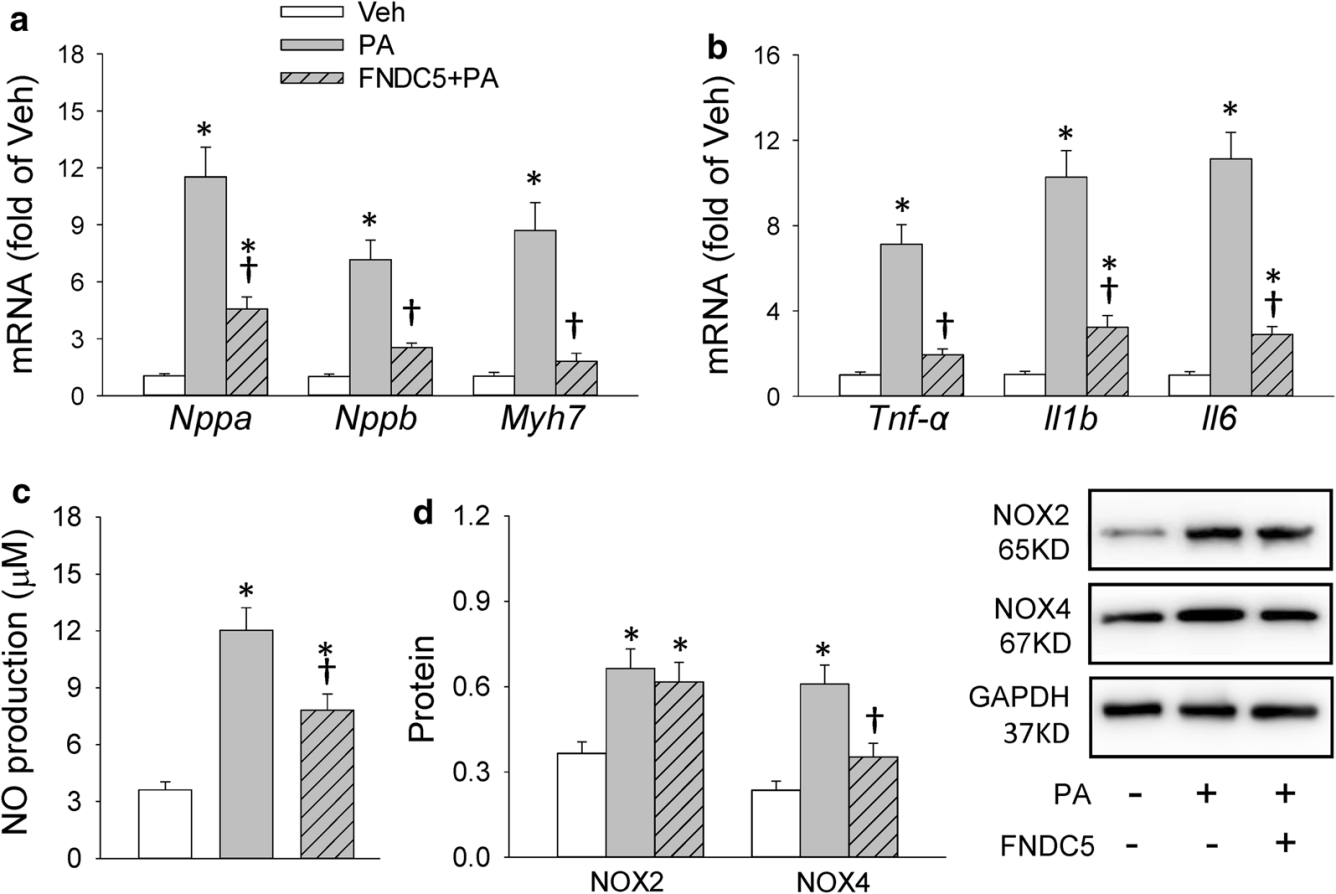
Exogenous FNDC5 pretreatment attenuated palmitate-induced inflammation and oxidative stress in CMs. **a**, **b** Effects of exogenous FNDC5 pretreatment on cardiac hypertrophy markers (*Nppa, Nppb* and *Myh7*) and inflammation markers (*Tnf*-*α, Il1b* and *Il6*) mRNA levels in PA-stimulated CMs. **c** Effects of exogenous FNDC5 pretreatment on NO production in cell culture supernatant of PA-stimulated CMs. **d** Effects of exogenous FNDC5 pretreatment on NOX expression in PA-stimulated CMs. PA: Palmitate; Veh: Vehicle. Values are mean ± SEM. *P < 0.05 vs. Veh. ^†^P < 0.05 vs. PA. n = 6

Since FNDC5 deficiency/knockdown aggravated inflammation in vitro, it is interesting to determine whether exogenous FNDC5 would play beneficial roles in attenuating PA-induced inflammation and oxidative stress in CMs derived from WT mice and in H9c2 cells. As shown in Fig. 5a, b, the up-regulated mRNA level of *Nppa, Nppb, Myh7, Tnf*-*α, Il1b* and *Il6* induced by PA were significantly decreased after exogenous FNDC5 pretreatment in CMs, indicating the cardiomyocyte hypertrophy-inhibitory effects and anti-inflammatory effects of FNDC5 in vitro. Also, the reduced NO production and NOX4 expression by exogenous FNDC5 pretreatment in PA-stimulated CMs showed anti-oxidative effects of FNDC5 (Fig. 5c, d). The similar effects of FNDC5 were observed in H9c2 cells (Additional file 1: Figure S4).

### FNDC5 attenuated cardiac hypertrophy, inflammation and oxidative stress in association with JAK2/STAT3 inhibition

Increasing evidence suggests that the JAK2/STAT3 pathway is involved in the development of cardiac hypertrophy [29, 30]. We firstly examined whether the phosphorylation of JAK2/STAT3 is disrupted by absence of FNDC5. As shown in Fig. 3c, HFD caused significant increases in phosphorylated JAK2 and STAT3 level in heart, and the enhancement were further improved in HFD-fed FNDC5^−/−^ mice. Similarly, we observed an increased phosphorylated JAK2 and STAT3 level in PA-stimulated H9c2 cells after knockdown of FNDC5 (Additional file 1: Figure S3C). Then we found WP1066, an inhibitor of JAK2 and STAT3, reversed exogenous FNDC5-induced cardiomyocyte hypertrophy-inhibitory effects, anti-inflammatory and anti-oxidative effects in PA-stimulated H9c2 cells (Additional file 1: Figure S4). Since both FNDC5 and WP1066 can inhibit JAK2/STAT3 signaling and thereby downregulate inflammation and oxidative stress, the reversed effects by WP1066 seems inconsistent. Moreover, we observed an increased phosphorylated JAK2/STAT3 level in WP + FNDC5 + PA group (Additional file 1: Figure S5). We speculated that maybe crosstalk between WP-related STAT3 signal and FNDC5/receptor downstream signaling antagonized the inhibited effects of WP and FNDC5 on JAK2/STAT3 signal, which leads to the augment of JAK2/STAT3 phosphorylation in WP + F+PA group. The augmented JAK2/STAT3 phosphorylation further contributes to the reversed effects by WP1066. Taken together, these results indicate that the effects of FNDC5 on cardiomyocytes are associated with JAK2/STAT3 inactivation.

### FNDC5 overexpression alleviated HFD-induced cardiac hypertrophy, inflammation and oxidative stress in mice

**Fig. 6:**
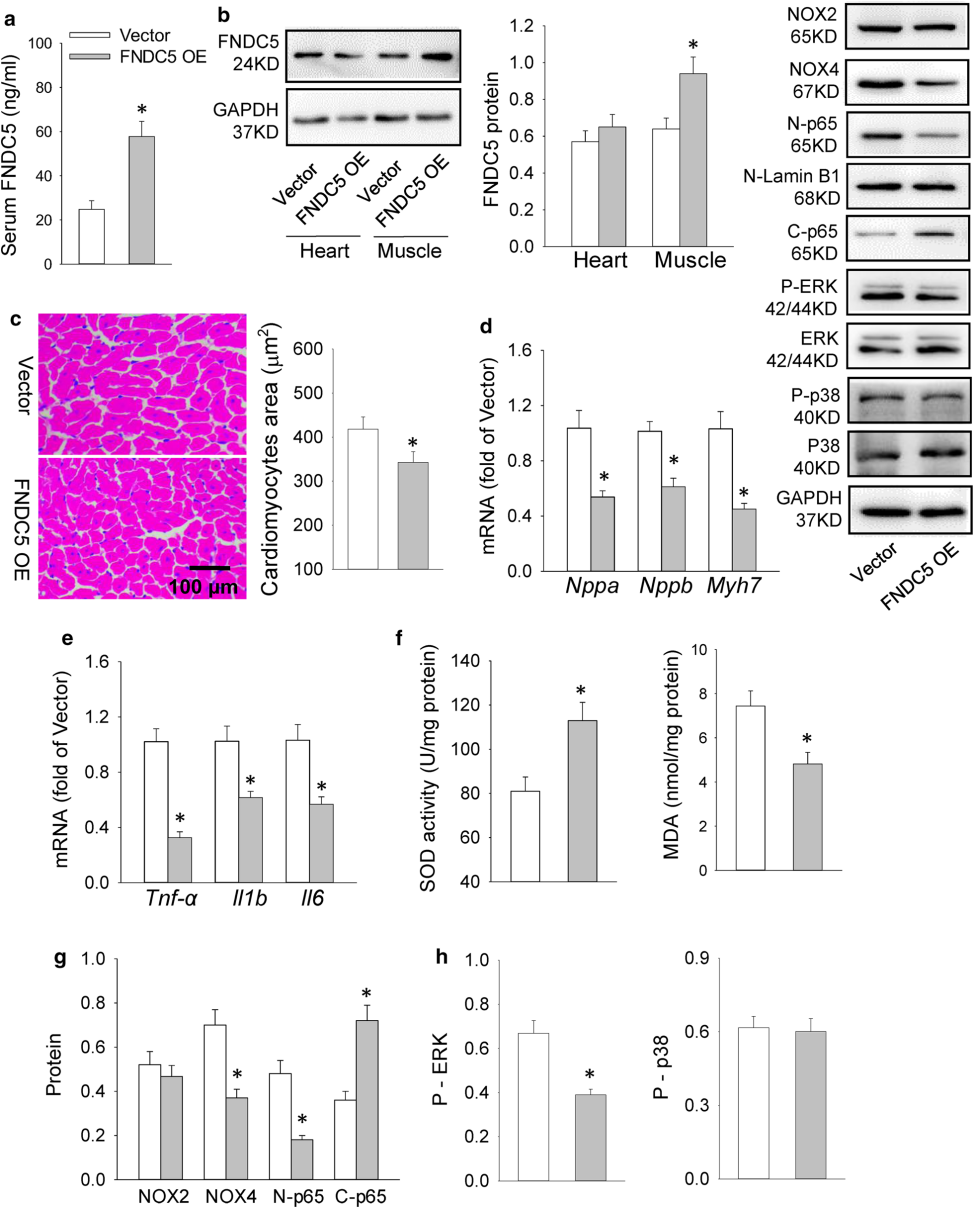
FNDC5 overexpression (OE) attenuated cardiac hypertrophy, inflammation and oxidative stress in HFD-fed mice. **a**, **b** Serum and heart/muscle FNDC5 levels after lentiviral vector-mediated FNDC5 overexpression. **c** Cardiomyocyte area in heart determined by H&E staining. **d**, **e** mRNA levels of the cardiac hypertrophy markers (*Nppa, Nppb* and *Myh7*) and inflammation markers (*Tnf*-*α, Il1b* and *Il6*). **f** SOD activity and MDA level in heart. **g** NOX2, NOX4 level and NFκB inactivation in heart. **h** Phosphorylation levels of p38 and ERK in heart. Values are mean ± SEM. *P < 0.05 vs. vector. n = 6

The effects of FNDC5 absence on cardiac inflammation further urged us to determine the possible therapeutic roles of FNDC5 in HFD-induced cardiac hypertrophy. Effectiveness of lentiviral vector-mediated FNDC5 overexpression was confirmed by the increased serum and muscle FNDC5 levels in mice (Fig. 6a, b), even it failed to increase FNDC5 expression in the heart (Fig. 6b). FNDC5 overexpression alleviated HFD-induced cardiac hypertrophy evidenced by reduced *Nppa, Nppb, Myh7* mRNA level and cardiomyocytes area (Fig. 6c, d). *Tnf*-*α, Il1b* and *Il6* mRNA level, NFκB activation, inflammatory signal (p-ERK) and oxidative stress markers (SOD activity and MDA level) as well as NOX4 expression were also decreased in FNDC5 overexpressed mice (Fig. 6e–h). As shown in Additional file 1: Figure S6, phospho-JAK2/STAT3 level were downregulated in FNDC5 OE mice, suggesting that the protective role of FNDC5 in cardiac inflammation are associated with inhibition of JAK2/STAT3 pathway.

## Discussion

Obesity has been recognized as the cause of various forms of cardiac abnormalities and development of heart failure [31, 32]. The primary novel findings in our study are that FNDC5 deficiency deteriorates, while FNDC5 overexpression attenuates HFD-induced cardiac hypertrophy, cardiac inflammation and oxidative stress. Supporting these in vivo observations, our in vitro studies also demonstrated that FNDC5 inhibits PA-induced cardiomyocytes hypertrophy via inactivated JAK2/STAT3 signaling-related inflammation and oxidative stress. Our results identify the protective role of FNDC5 in obesity-induced cardiac hypertrophy, which shed additional light on future FNDC5-based therapeutic interventions in obesity-related cardiac abnormalities.

Low grade inflammation serves as a proximate trigger, as well as an ultimate modulator of cardiac dysfunction in obesity and cardiovascular disease [33, 34]. Increased oxidative stress coupled with activation of down-stream pro-inflammatory cytokines have pivotal roles in the development of obesity-related complications [35, 36]. As we known, lipid overload is often associated with increased production and release of pro-inflammatory cytokines, and these cytokines activate a series of intracellular signaling pathways including NFκB, which up-regulate the transcription of more cytokines and exaggerate the inflammatory response. These events further trigger macrophage activation and tissue filtration, leading to tissue injury [37]. According to our data, FNDC5 gene deletion not only exacerbated HFD-induced enhancement of the pro-inflammatory cytokines levels, oxidative stress and NOX4 expression in heart, but also promoted NFκB activation and cardiac inflammatory signaling activation. The anti-inflammatory effects by FNDC5 were not only demonstrated in heart of HFD-fed mice, but also in PA-stimulated CMs and H9c2 cells. These data confirm the strong anti-inflammatory effects of FNDC5 on cardiomyocytes, which may contribute to its cardio-protective effects in the present study.

To support the direct role of fatty acid (FA) in cardiomyocyte hypertrophy, in vitro exposure of cardiomyocytes to PA was investigated in our study. Both CMs and H9c2 cells under high lipid treatment showed upregulated inflammatory responses as well as oxidative stress levels, which were also attenuated by exogenous FNDC5 pretreatment, indicating that FNDC5 is involved in the cardio-protective effects against PA-induced inflammation and oxidative stress in vitro. It has been shown that FNDC5 overexpression facilitates the process of cardiogenesis [38]. A recently study found that irisin, the secreted protein derived from FNDC5, protects heart against ischemia–reperfusion injury through a mitochondria mechanism [39]. Although previous studies reported some evidences of the positive role of FNDC5 in heart, the exact role in inflammation and oxidative stress on cardiomyocytes were first investigated in our present study.

According to our in vitro and in vivo data, FNDC5 absence/knockdown led to an enhanced phosphorylation level of JAK2 and STAT3 under high lipid treatment (HFD/PA) accompanied by aggravated inflammation, oxidative stress and cardiac hypertrophy. Phospho-JAK2/STAT3 level were downregulated in FNDC5 OE mice, suggesting that the protective role of FNDC5 in cardiac inflammation are associated with JAK2/STAT3 pathway inhibition. As we known, obesity results in marked alterations in cardiac energy metabolism, with a prominent effect being an increase in fatty acid uptake and oxidation by the heart [40]. Peroxisome proliferator activator receptor (PPAR)-α is expressed in abundant levels in the heart and enhanced PPAR-α signaling is especially associated with increased fatty acid oxidation in cardiomyocytes. Interestingly, PPAR-α acts downstream of FNDC5 [9]. We recently found that FNDC5 deficiency causes impairment in AMPK/PPAR-α-mediated fatty acid oxidation in hepatocytes [11]. However, it is unclear whether FNDC5 is involved in cardiac fatty acid oxidation during obesity, and the crosstalk between PPAR-α and JAK2/STAT3 signaling or other signal transduction molecules needs further investigation. Elucidation of the precise molecular mechanisms underpinning FNDC5 signaling involved in the cardiac hypertrophy process may prove useful in understanding the role of FNDC5 in metabolic-related cardiovascular diseases.

Adipose tissue inflammation induce systemic inflammation and insulin resistance, resulting in subcellular low-grade inflammation, and in turn, subcellular component abnormalities such as oxidative stress, mitochondrial dysfunction in the heart. The vicious cycle of increased subcellular component abnormalities, inflammatory cell infiltration, neurohumoral activation induce the adverse cardiac remodeling in metabolic-induced cardiomyopathy. Taken together the findings of our present and previous studies, FNDC5 not only ameliorated systemic inflammation, but also had direct effects on cardiomyocytes, which may both contribute to the cardio-protective actions. Although it is still difficult to unmask the probable effectors (receptors) of endogenous FNDC5 and its cleaved fragment, irisin, we considered that FNDC5 may exert its protective function through both direct and indirect pathways. Our data show that HFD did not lead to any significant change in FNDC5 expression level in heart. Similarly, FNDC5 overexpression only increased circulating and muscle expression but not FNDC5 in heart. It is possible that, circulating but not cardiac-derived FNDC5 contributes to the beneficial and cardio-protective effects in vivo.

Skeletal muscle can act as an endocrine organ, secreting diverse myokines in a context-dependent manner [41, 42]. Such myokines may exert specific endocrine effects on visceral fat, or work locally within the muscle and exert their effects on signaling pathways involved in fat oxidation and glucose uptake, contributing to its beneficial effects on obesity and related diseases. Sundarrajan et al. showed that exogenous irisin, the cleaved fragment of FNDC5, increased diastolic volume, heart rate and cardiac output in zebrafish by regulating cardiovascular physiology-associated modulators such as myostatin a/b, troponin C expression [43]. Also, it has been reported that irisin plays a protective role against ischemia–reperfusion injury to heart through a SOD2-dependent mitochondria mechanism [39]. These studies indicate significant translational value of myokines for therapeutic approaches in cardiovascular diseases. Our study highlights the important myokines as novel targets of FNDC5/irisin. Future studies will be required to elucidate the secretory and metabolic pathways for regulation of FNDC5/irisin levels in circulation, which may present significant translational value for clinical implications.

## Conclusions

**Fig. 7:**
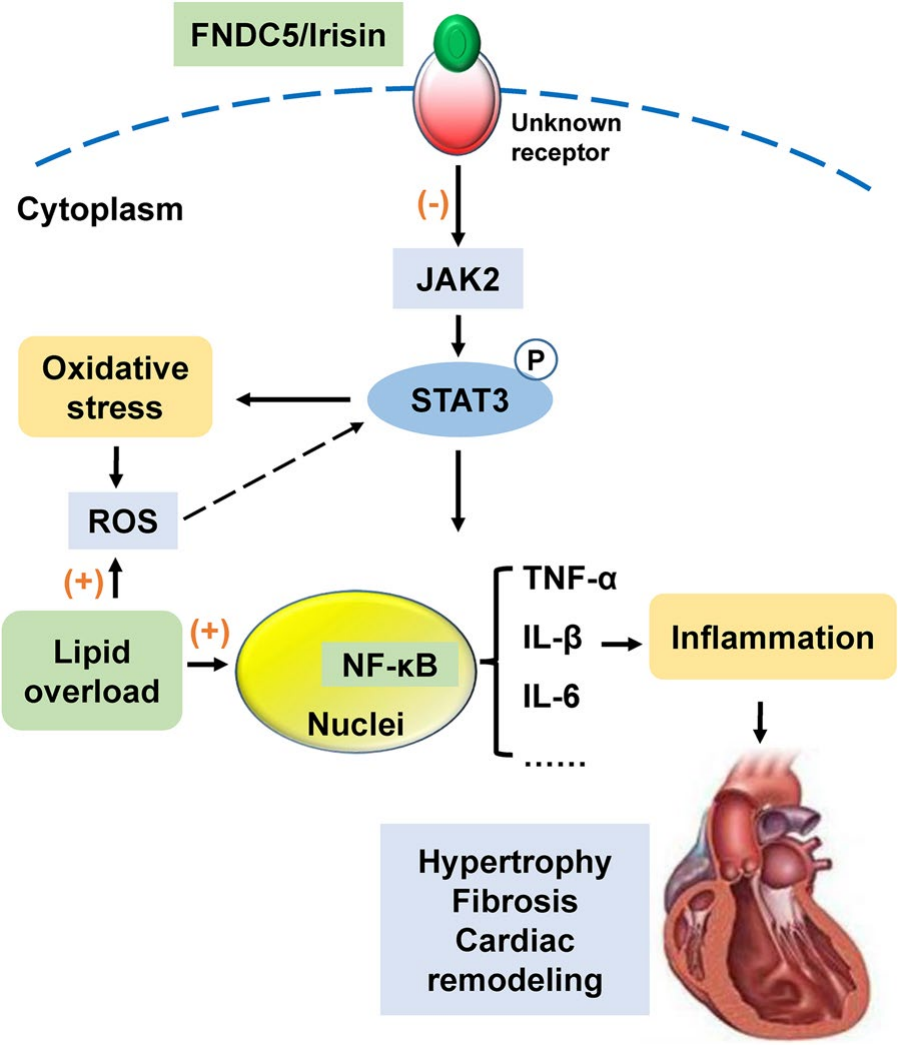
Schematic illustrates a possible mechanism of FNDC5 in obesity-induced cardiac hypertrophy. Obesity-induced lipid overload has a great impact on the production of reactive oxygen species (ROS) and upregulation of TNF-α, IL-1β and IL-6 levels, which leads to cardiac inflammation and oxidative stress. The enhanced cardiac inflammation and oxidative stress are involved in pathogenesis of cardiac injury and remodeling. FNDC5 may exert its protective function on the enhanced inflammation and oxidative stress via JAK2/STAT3 pathway through an unknown receptor

FNDC5 ameliorated HFD-induced cardiac hypertrophy, via the effects on inflammation, oxidative stress and NFκB activation, in association with JAK2/STAT3 signaling inhibition (Fig. 7). The cardio-protective role of FNDC5 servers for future therapeutic interventions in obesity-related cardiac complications.

## Additional file

**Additional file 1: Table S1.** Primers used for real-time PCR. **Figure S1**. FNDC5 deficiency aggravated HFD-induced cardiac hypertrophy and enhanced cardiac TNF-α, IL-1β and IL-6 levels in mice. **Figure S2**. FNDC5 deficiency enhanced NLRP3 and IL18 mRNA levels in heart of mice. **Figure S3**. Knockdown of FNDC5 enhanced palmitate-induced inflammation and NOX4 expression in H9c2 cells. **Figure S4**. Effects of exogenous FNDC5 and JAK2/STAT3 inhibitor pretreatment on inflammation and oxidative stress in H9c2 cells. **Figure S5**. Effects of exogenous FNDC5 and JAK2/STAT3 inhibitor pretreatment on phosphorylated JAK2 and STAT3 level in H9c2 cells. **Figure S6**. FNDC5 overexpression attenuated phosphorylated JAK2 and STAT3 level in heart of HFD-fed mice.

## Abbreviations

FNDC5: fibronectin type III domain containing 5
HFD: high fat diet
Nppa: natriuretic peptide type A
Nppb: natriuretic peptide type B
Myh7: myosin, heavy polypeptide 7, cardiac muscle, beta
Tnfɑ: tumor necrosis factor ɑ
Il1b: interleukin 1 beta
Il6: interleukin 6
Il18: interleukin 18
Nlrp3: NLR family, pyrin domain containing 3
PA: palmitate
JAK2: Janus kinase 2
STAT3: signal transducer and activator of transcription 3
NOX: nicotinamide adenine dinucleotide phosphate oxidase
SOD: superoxide dismutase
MDA: malondialdehyde
