# Supplementary material for: FNDC5 attenuates obesity-induced cardiac hypertrophy by inactivating JAK2/STAT3-associated inflammation and oxidative stress
Source: J Transl Med. 2019 Apr 2;17:107. doi: 10.1186/s12967-019-1857-8 (PMC6444535; doi:10.1186/s12967-019-1857-8)
Supplement: Supplementary file 1 — Additional file 1: Table S1. Primers used for real-time PCR. Figure S1. FNDC5 deficiency aggravated HFD-induced cardiac hypertrophy and enhanced cardiac TNF-α, IL-1β and IL-6 levels in mice. Figure S2. FNDC5 deficiency enhanced NLRP3 and IL18 mRNA levels in heart of mice. Figure S3. Knockdown of FNDC5 enhanced palmitate-induced inflammation and NOX4 expression in H9c2 cells. Figure S4. Effects of exogenous FNDC5 and JAK2/STAT3 inhibitor pretreatment on inflammation and oxidative stress in H9c2 cells. Figure S5. Effects of exogenous FNDC5 and JAK2/STAT3 inhibitor pretreatment on phosphorylated JAK2 and STAT3 level in H9c2 cells. Figure S6. FNDC5 overexpression attenuated phosphorylated JAK2 and STAT3 level in heart of HFD-fed mice. [file 12967_2019_1857_MOESM1_ESM.docx]

Additional Material

FNDC5 attenuates obesity-induced cardiac hypertrophy by inactivating JAK2/STAT3-associated inflammation and oxidative stress

Zhi Geng, Wen-Yong Fan, Bing Zhou, Chao Ye, Ying Tong, Ye-Bo Zhou, Xiao-Qing Xiong*

**Table S1**—Primers used for real-time PCR.


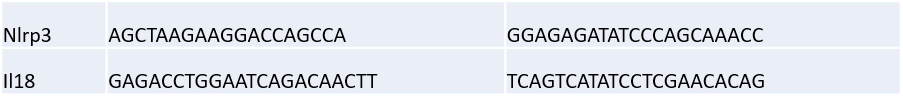

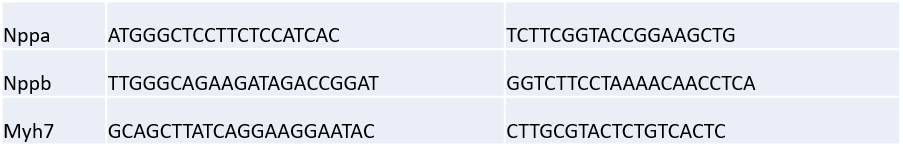

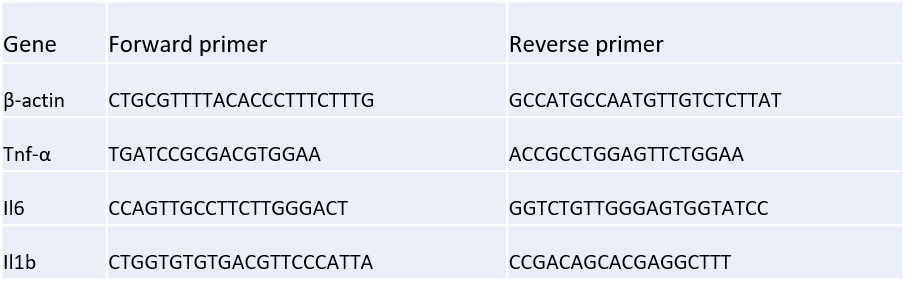


**
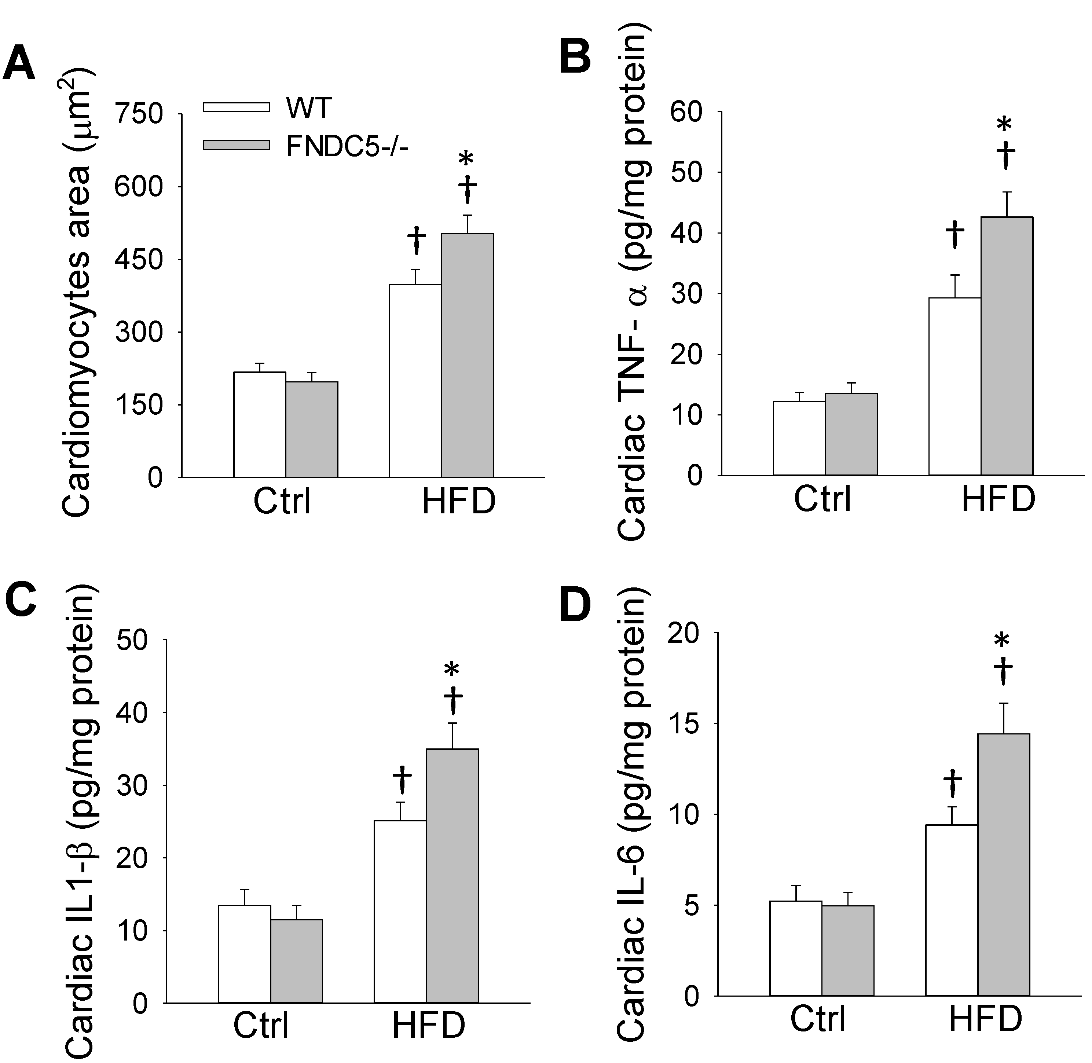
**

**Figure S1. FNDC5 deficiency aggravated HFD-induced cardiac hypertrophy and cardiac inflammation in mice.** WT and FNDC5^−/−^ mice were fed with mouse chow diet (Ctrl) and high fat diet (HFD) for 20 weeks.(A) Cardiomyocyte area in heart tissue of mice. (B-D) Expression of TNF-α, IL-1β and IL-6 levels in heart determined with ELISA. Values are mean ± SEM. *P<0.05 vs. WT. †P<0.05 vs. Ctrl. n=6.

**
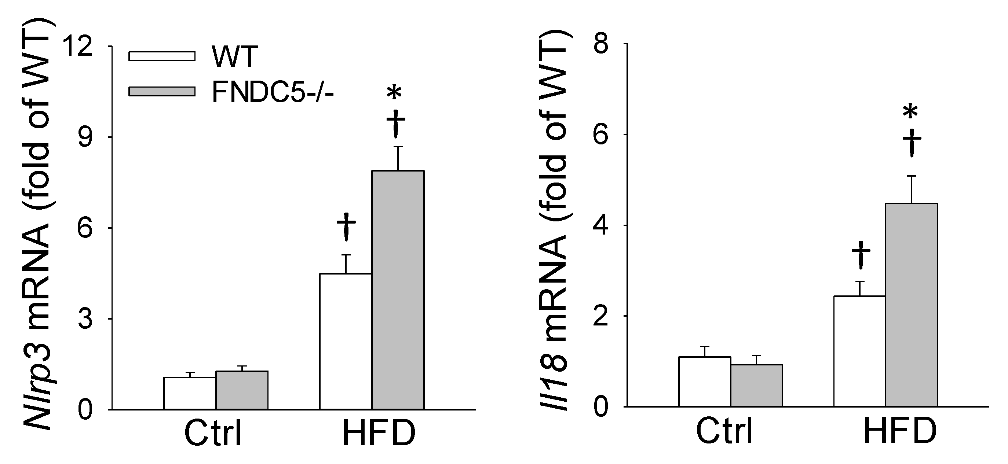
**

**Figure S2. Effects of FNDC5 deficiency on NLRP3 and IL18 mRNA in heart of mice.** WT and FNDC5^−/−^ mice were fed with mouse chow diet (Ctrl) and high fat diet (HFD) for 20 weeks. Values are mean ± SEM. *P<0.05 vs. WT. †P<0.05 vs. Ctrl. n=3.

**
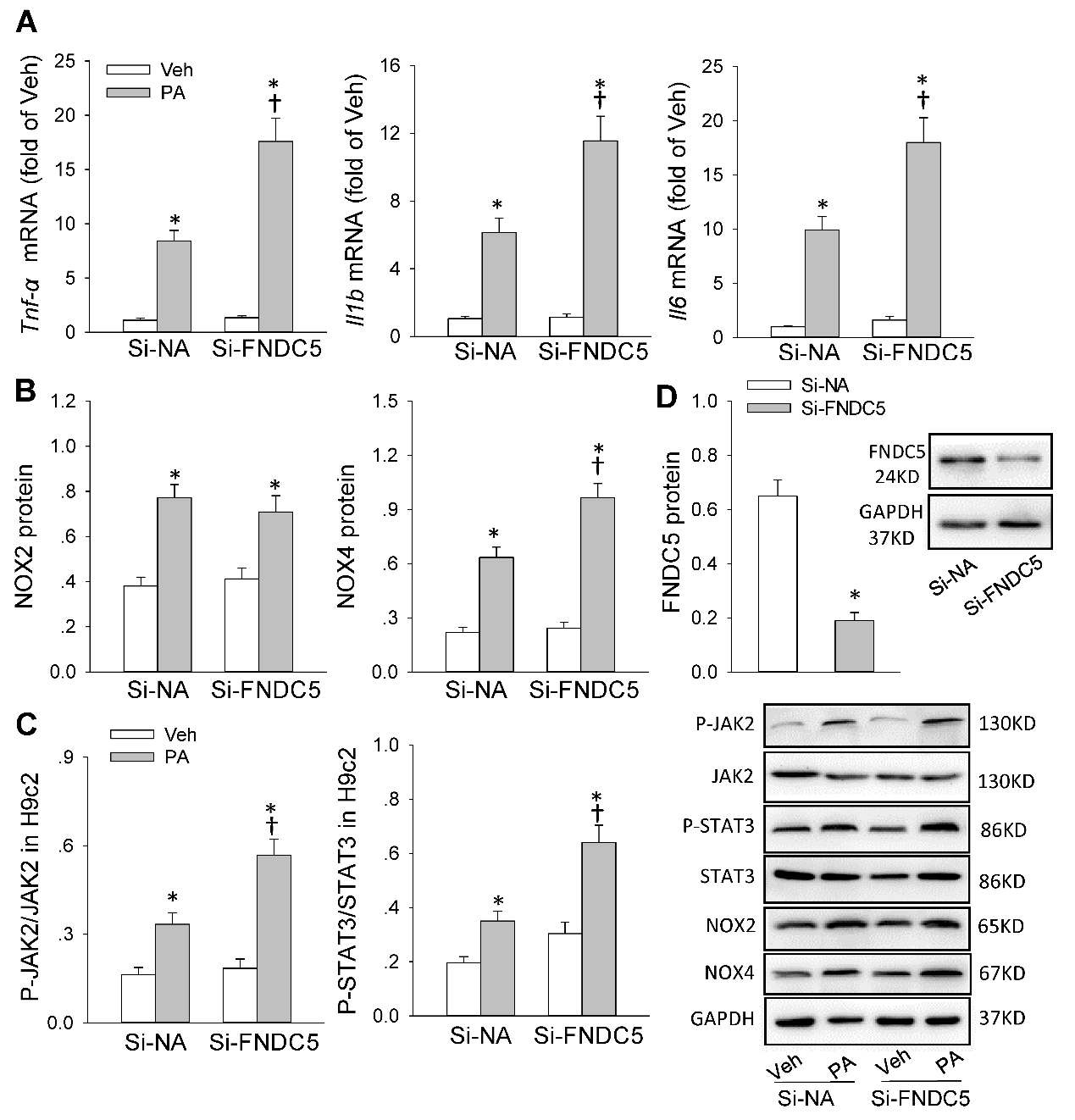
**

**Figure S3. Knockdown of FNDC5 enhanced palmitate-induced inflammation and NOX4 expression in H9c2 cells.** (A) Effects of FNDC5 siRNA (Si-FNDC5) or NA siRNA (Si-NA) for 48 h (50 nM) on TNF-α, IL-1β and IL-6 mRNA levels with or without palmitate (PA, 400 μM) treatment. (B) Effects of siRNA on NOX2 and NOX4 protein level with or without PA treatment. The measurement was 24 h after PA treatment for measuring the mRNA levels or protein levels. (C) Effects of siRNA on phosphorylated JAK2 and STAT3 level. The measurement was made 12 h after PA administration. (D) Effects of siRNA on FNDC5 protein expression. PA: Palmitate; Veh: Vehicle. Values are mean ± SEM. In (A-C), *P<0.05 vs. Veh. †P<0.05 vs. Si-NA. In (D), *P<0.05 vs. Si-NA. n=3.

**
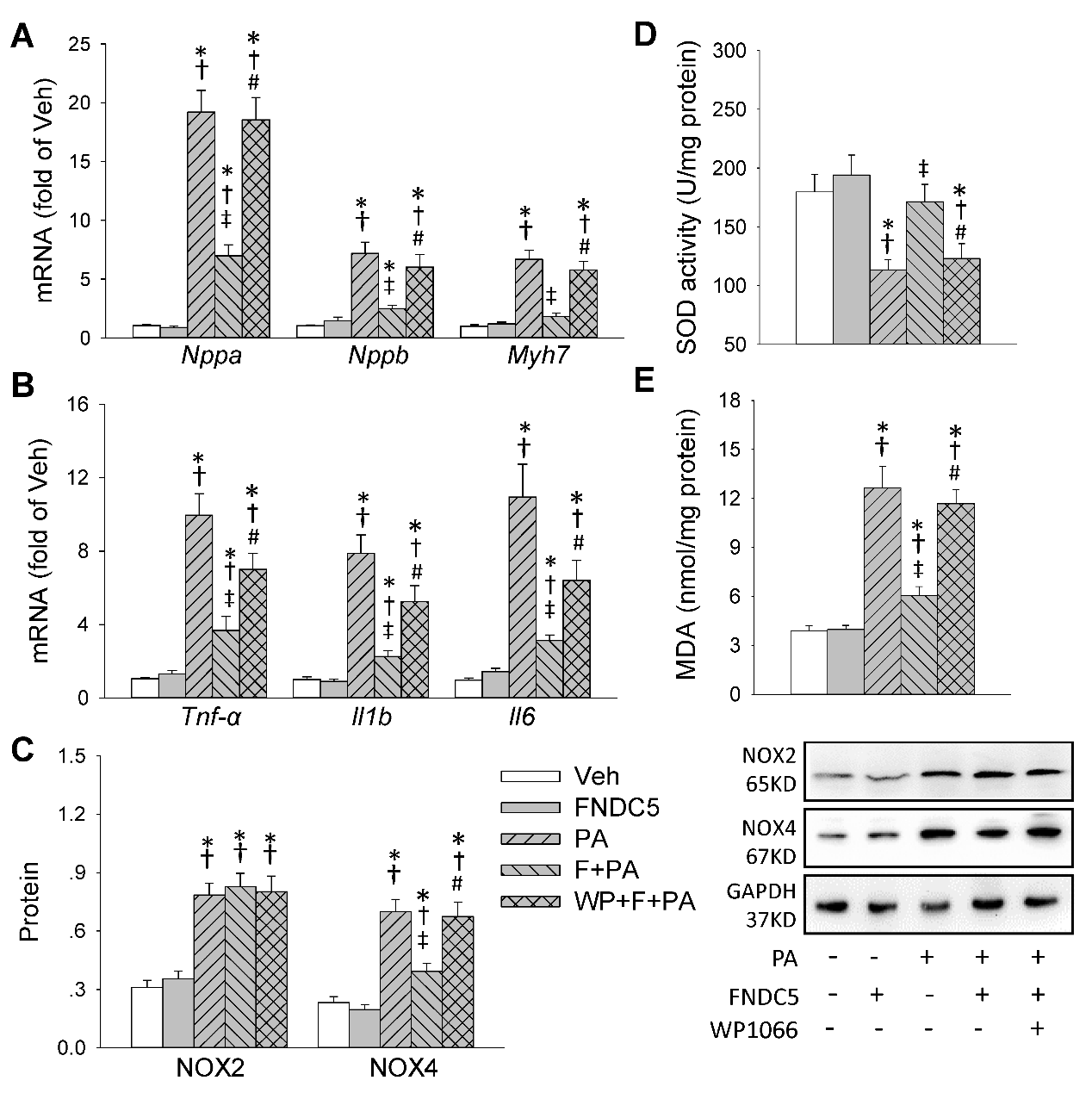
**

**Figure S4**. **Effect of exogenous FNDC5 and JAK2/STAT3 inhibitor pretreatment on inflammation and oxidative stress in H9c2 cells.** (A, B) Effects of JAK2/STAT3 inhibitor WP1066 (WP) and/or FNDC5 pretreatment on cardiac hypertrophy markers (*Nppa, Nppb, Myh7*) and inflammation markers (*Tnf-α, Il1b* and *Il6*) mRNA levels in H9c2 cells. (C, D, E) Effects of WP and/or FNDC5 pretreatment on SOD activity, MDA level and NOX2, NOX4 protein levels in H9c2 cells. PA: Palmitate; Veh: Vehicle; F: FNDC5. PA was administrated 4 h after exogenous FNDC5 (200 nM) or FNDC5 combined WP (5 μM) pretreatment. The measurement was made 24 h after PA administration. Values are mean ± SEM. *P<0.05 vs. Veh; †P<0.05 vs. FNDC5; ‡ P<0.05 vs. PA; # P<0.05 vs. FNDC5+PA. n=3.





**Figure S5**. **Effects of exogenous FNDC5 and JAK2/STAT3 inhibitor pretreatment on** **phosphorylated JAK2 and STAT3 level in H9c2 cells.** PA: Palmitate; Veh: Vehicle; F: FNDC5. PA was administrated 4 h after exogenous FNDC5 (200 nM) or FNDC5 combined WP (5 μM) pretreatment. The measurement was made 12 h after PA administration. Values are mean ± SEM. *P<0.05 vs. Veh; †P<0.05 vs. FNDC5; ‡ P<0.05 vs. PA; # P<0.05 vs. FNDC5+PA. ^P<0.05 vs. WP; $ P<0.05 vs. WP+PA. n=3.

**
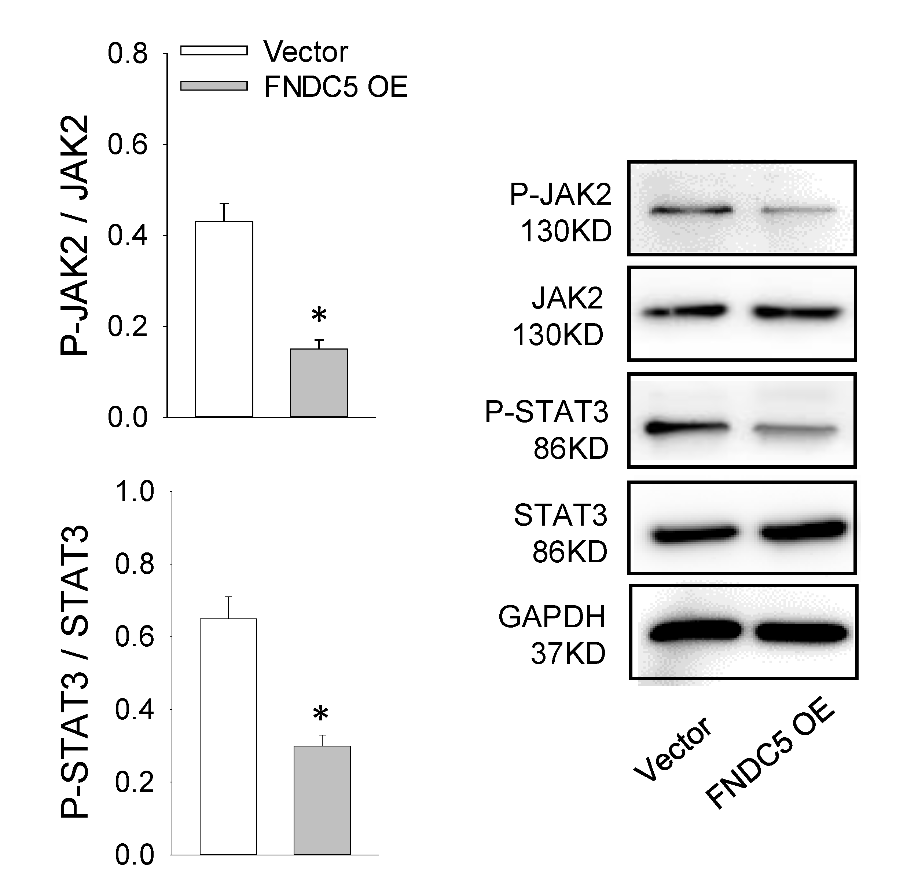
**

**Figure S6. FNDC5 overexpression (OE) attenuated phosphorylated JAK2 and STAT3 level in heart of HFD-fed mice.** The recombinant lentivirus expressing FNDC5 or vector were injected into the mice at the end of the 14th week after HFD. The measurements were carried out 6 weeks after the lentivirus injection. Values are mean ± SEM. *P<0.05 vs. vector. n=3.
